# Antibacterial Activity of Essential Oils from Palmarosa, Evening Primrose, Lavender and Tuberose

**DOI:** 10.4103/0250-474X.54278

**Published:** 2009

**Authors:** M. H. Lodhia, K. R. Bhatt, V. S. Thaker

**Affiliations:** Department of Biosciences, Saurashtra University, Rajkot-360 005, India

**Keywords:** Antibacterial activity, gram-positive bacteria, gram-negative bacteria, essential oil, evening primrose, lavender, palmarosa, tuberose

## Abstract

Essential oils extracted from flower petals of palmarosa (*Cymbopogon martini*), evening primrose (*Primula rosea*), lavender (*Lavandula angustifolia*) and tuberose (*Polianthus tuberosa*) were tested for their antibacterial activities against gram-positive and gram-negative bacteria. Different concentrations of each essential oil ranging from 10-100% were tested. Both gram-positive and gram-negative bacteria were found susceptible to the studied flower essential oils. With increase in concentration of essential oil, increase in zone of inhibition was observed thus dose-dependent response was clear for each essential oil. Essential oil extracted from *Cymbopogon martini* showed the highest activity against both gram positive and gram negative bacteria among the tested essential oils.

Volatile compounds from plants, especially essential oils have antimicrobial, fungicidal and insecticidal activities[1,2]. The essential oils are strong antimicrobial agents with broad spectrum activity with possible potential for the control of pathogens in plants as of post-harvest spoilage of many crops and also to human pathogenic diseases[3]. They may prove to be more economical and environmentally safe, as an antimicrobial agent.

The volatile essential oils released from leaves, flowers and fruits into the atmosphere and from roots into the soil defend herbivores and pathogens[4]. Essential oil from flower petals of Palmarosa is used for gargles in throat infection, and skin care[2]. Evening primrose is well known for skin care and beauty treatments in Ayurvedic medicines[5]. Tuberose is an ornamental plant, which is widely used in aeromatherapy because of its flavour and fragrance[6]. Lavender essential oil treats sinus and vaginal infection, including candida, and useful as an excellent treatment for laryngitis and asthma. It relieves muscle pain, headaches, insect bites, cystitis and other inflammation. Essential oil of lavender also treats digestive disturbances including colic and helps to boost immunity[7].

Plant essential oils constitute about 1% of plant secondary metabolites and are mainly represented by terpenoids, phenylpropanoids or benzenoids, fatty acid derivatives and amino acid derivatives[4]. Earlier workers have isolated some potent constituents like geraniols (terpene alcohol) from palmarosa[8], triterpenoidsaponin (primulanin) in evening primrose[7], lavandin[5], linalyl actate, geraniol (terpene alcohol) and citronellal[9] from lavender. Tuberose leaves possess kaempferol and tuberolide[10]. Due to low molecular weight with lipophilic tendencies, essential oils have the capacity to penetrate into the tissue easily and much faster. Only scattered attempts have been made to demonstrate the ability of essential oils in antimicrobial and antiherbivore activity[11,12].

Out of two selected organisms, *Staphylococcus aureus* causes a variety of suppurative infections in humans. It causes superficial skin lesions; more serious infections such as pneumonia, meningitis, and urinary tract infections; food poisoning by releasing enterotoxins into food and toxic shock syndrome by release of superantigens into the blood stream. *Escherichia coli* is normally present in human intestine but eventually cause food poisoning. The prominent difference between *E. coli* and *S. aureus* is their gram staining property. *E. coli* is gram positive while *S. aureus* is gram negative bacteria. The present work was carried out to find out the common and potent agent that can inhibit the growth of both types of bacteria.

Steam distilled essential oils from flowers of palmarosa, evening primrose, tuberose and lavender were obtained from VIRSACO (Vimal Research Society for Agrobiotech and Cosmic Power), Rajkot, Gujarat, India. In the present study, test organisms *E. coli* (2006) was obtained from NCIM, Pune, India and *S. aureus* (V001) from VIRSACO, Rajkot, India, respectively. Antibacterial activity was performed using standard disc diffusion method described by Rastogi and Mehrotra[13]. Bacterial suspension (100 μl), containing 6×10^6^ cfu/ml of *S. aureus* and *E. coli* was spread on Petri dishes (9 cm in diameter) homogenously with the help of glass spreader in aseptic condition. Paper discs were dipped in the different concentrations of essential oils ranging from 10-100% and kept in the centre of Petri dishes. Dilutions of essential oils were prepared with juniper oil using as base oil because it did not show activity against these two organisms. The plates were incubated for 24 h at room temperature. After 24 h incubation, the diameter of clear zone surrounding the disc was measured in mm. The experiment was repeated twice with three replicates for each concentration of all the essential oils.

The steam distilled extract of the flowers showed marked activity against both gram-positive and gram-negative bacteria (Table 1). The essential oils from different plants species tested were found more effective at very low concentration even for antifungal activity[14]. The disc absorption capacity was 5 μl/disc only. The results noted in the Table 1 showed the dose-dependent response for all four plants used. Essential oil of palmarosa exhibited potent antibacterial activity among all the essential oils tested. Essential oil of lavender and palmarosa showed potent effect on gram negative organism in all the concentrations tested. Tuberose showed more influence at lower concentration on gram negative than gram positive bacteria. In evening primrose lower concentration has more influence on gram negative however; higher concentration showed distinct effect on gram positive bacteria. It is reported that gram positive bacteria are more susceptible to essential oils than gram negative bacteria[15,16]. The weak antibacterial activity against the gram negative bacteria was ascribed to the presence of an outer membrane that possess hydrophilic polysaccharides chains as a barrier for hydrophobic essential oils[17]. Contrary to this, selected essential oils showed equal competence to both gram positive bacteria as well as gram negative bacteria. In conclusion, essential oils are potential agent against both gram negative and gram positive bacteria. Similar experimentations can help to explore the potential role of essential oils as antibacterial agents but requires further study.

**TABLE 1:** ANTIBACTERIAL ACTIVITY OF PLANT ESSENTIAL OILS

Essential oils	Organisms	Zone of inhibition against various concentrations of essential oil (in mm)			
		
		10%	25%	50%	100%
*Lavandula angustifolia*	*E. coli*	10.5	10.6	11.2	17.5
	*S. aureus*	6.0	6.5	9.0	13.0
*Cymbopogon martini*	*E. coli*	12.2	16	-	29.7
	*S. aureus*	6.5	8.0	18.0	22.0
*Polianthus tuberose*	*E. coli*	8.06	-	12.5	15.0
	*S. aureus*	6.0	6.5	7.0	15.0
*Primula rosea*	*E. coli*	10.1	13.0	18.0	21.5
	*S. aureus*	6.0	7.0	11.0	28.0

Antibacterial activity of plant essential oils against gram-positive and gram-negative bacteria. Results are expressed as the mean value of six replicates
